# Trends in Hospitalization of Patients with Potentially Serious Diseases Evaluated at a Quick Diagnosis Clinic

**DOI:** 10.3390/diagnostics10080585

**Published:** 2020-08-13

**Authors:** Xavier Bosch, Andrea Ladino, Pedro Moreno-Lozano, Anna Jordán, Alfonso López-Soto

**Affiliations:** Department of Internal Medicine, Institut d’Investigacions Biomèdiques August Pi i Sunyer (IDIBAPS), University of Barcelona, Hospital Clínic, 08036 Barcelona, Spain; Ladino@clinic.cat (A.L.); pjmoreno@clinic.cat (P.M.-L.); aijordan@clinic.cat (A.J.); alopez@clinic.cat (A.L.-S.)

**Keywords:** trends in hospitalization, quick diagnosis units, diagnosis, hospital ambulatory medicine, cancer, anemia, invasive procedures, performance status, daycare center

## Abstract

Although quick diagnosis units (QDU) have become a cost-effective alternative to inpatient admission for diagnosis of potentially serious diseases, the rate of return hospitalizations among evaluated patients is unknown. This study examined the temporal trends in admissions of QDU patients through 15 years. Adult patients referred to QDU from 2004 to 2019 who were hospitalized between the first and last visit in the unit were eligible. Decisions about admissions were mainly based on the Appropriateness Evaluation Protocol and required independent validation by experienced clinicians using a customized tool. The final analysis included 825 patients. Patient characteristics and major reasons for admission were compared each year and linear trends were analyzed. Admission rates decreased from 7.2% in 2004–2005 to 4.3% in 2018–2019 (*p* < 0.0001). While a significant increasing trend was observed in the rate of admissions due to cancer-related complications (from 39.5% in 2004–2005 to 61.7% in 2018–2019; *p* < 0.0001), those due to anemia-related complications and scheduled invasive procedures experienced a significant downward trend. A likely explanation for these declining trends was the relocation of the unit to a new daycare center in 2013–2014 with recovery rooms and armchairs for IV treatments. The facts of this study could help in the provision of anticipatory guidance for the optimal management of patients at risk of clinical complications.

## 1. Introduction

The recognition 15 years ago that excessive healthcare expenses, especially hospital care expenses, could be reduced by reducing the number of hospital beds and staff motivated the search for alternative strategies to avoid the need for inpatient admission [1,2,3]. The term ‘hospital ambulatory medicine’ was recently coined to denote a shift from traditional ‘bed-based’ inpatient care to hospital-based ambulatory and outpatient care services [4]. These alternative management models for medical conditions conventionally designed to require hospitalization include, among others, daycare hospitals, hospital-at-home, observation units, outpatient management, and quick diagnosis units [5,6,7,8,9,10,11].

By avoiding inpatient admissions for purely diagnostic purposes, quick diagnosis units have become a different care delivery approach specifically designed to achieve rapid diagnoses for patients with potentially serious illnesses, especially cancer, referred from outpatient settings or the emergency department [10]. The concept of ‘quick diagnosis units’ as ambulatory care clinics led by general internists, which do not involve an overnight or multi-day stay in hospital, was originally developed and reported by Spanish authors in the 2000s and early 2010s [10,12,13,14,15]. Different, mainly observational studies revealed that these units were as clinically effective as inpatient admission to reach a diagnosis, with similar diagnostic times. While patient reported satisfaction was also similar or higher than conventional hospitalization, quick diagnosis units proved cost-effective with associated costs significantly lower than the same costs applied to the evaluation of the same conditions in the hospital setting [16,17,18,19,20,21,22,23,24,25].

In a recent position report of the European Federation of Internal Medicine, Corbella et al. supported the implementation of innovative hospital ambulatory care strategies including quick diagnosis units [4]. In their report, the authors argued that current health care issues should be approached with a perspective of changing the hospital model, moving from volume-based care to value-based care models where health outcomes can be measured easily.

A new care delivery model with high-quality standards would be expected to be associated with a low number of unwanted outcomes or quality indicators related to patient safety, mortality, or readmissions. Although the evidence is limited, the quick diagnosis unit model has been reported to be safe and associated with low mortality rates. However, it is not well known whether the diagnostic process in these units is associated with return hospitalizations or unwanted admissions of evaluated patients.

With the purpose of addressing this issue, the current study investigated the temporal trends and causes of hospitalizations of patients with potentially severe illnesses who were managed at a hospital-based quick diagnosis unit during a 15 year period.

## 2. Materials and Methods

### 2.1. Setting

The structure, indications for referral, and operating procedures of the quick diagnosis unit have been reported previously [10,19]. In short, this unit is an ambulatory or outpatient care clinic of the Internal Medicine Department of the Hospital Clínic of Barcelona, a third-level university hospital with a reference population of about 550,000. The unit first operated as a traditional outpatient clinic from 2004 to 2013 to be then transferred to the adult daycare center which became its definitive location. Patients are principally referred from the hospital emergency department and 15 primary care centers. As a requirement for referral, patients’ general condition must be acceptable to enable them to go to hospital for investigations and appointments, then back to home. Most disorders are evaluated based on evidence-based diagnostic protocols and clinical practice guidelines. Investigations and procedures differ according to the indication for referral and are carried out as appropriate according to the clinical evaluation during the first and successive appointments.

### 2.2. Study Design and Population

In this retrospective observational study, all consecutive patients aged >18 years referred to the quick diagnosis unit from 2004 to 2019 who were hospitalized after the first visit in the unit until the date of completion of the evaluation process were assessed for eligibility. The primary outcome was hospitalization. Patients whose hospitalization was judged as inappropriate by the panel of independent reviewers (see below) were excluded. The research ethics committee of the Hospital Clínic gave approval to the study and all patients were required to approve a written informed consent for their enrollment (approval code 0716, date: 13 December 2019). The ethical guidelines of the Declaration of Helsinki were also observed.

Patients referred to the unit were managed at the discretion of its physicians, comprising of a staff attending physician (a consultant internist) and senior residents of internal medicine. Clinical decisions regarding admissions were made on a case by case basis with consideration of different patient factors such as severity of illness, refractory pain, persistent fever, or added multimorbidity. Decisions about admissions were mainly based on the diagnosis-independent Appropriateness Evaluation Protocol (AEP), which describes the criteria for appropriateness of admissions including reasons for inappropriate admissions of adult patients at internal medicine, surgery, and gynecology inpatient facilities [26]. Under criteria contemplated in the European version of AEP, for instance, admissions to avoid the waiting time for an outpatient investigation would be deemed as inappropriate [27]. However, an admission for ‘performing a biopsy of an internal organ’ is deemed as an appropriate admission. For an admission to be deemed appropriate, at least one of 16 AEP criteria must be met.

Inclusion in the study required independent validation by three experienced clinicians of our hospital, who reviewed the medical records of all patients hospitalized for the duration of the study. The appropriateness of admission decisions was determined according to disease severity and required level of care, which are the main AEP dimensions. For our study purpose, a customized tool was created based on the Spanish [26], European [27], German [28], and original US versions of AEP [29]. This tool included criteria for appropriateness of admissions, justification of appropriateness, and reasons for inappropriateness (Figure S1). The physician reviewers were trained before the study started and we held periodic meetings to resolve disputes. All the reviews were performed independently and concurrently. We measured the degree of agreement between the three reviewers regarding the appropriateness and inappropriateness of admission decisions. The overall inter-rater agreement was assessed using Cohen’s kappa (κ) statistic, which was interpreted as set out by Landis and Koch [30]. According to these guidelines, kappa coefficients between 0.00–0.20 indicate slight agreement, between 0.21–0.40 fair agreement, between 0.41–0.60 moderate agreement, between 0.61–0.80 substantial agreement, and between 0.81–1.00 an almost perfect agreement.

### 2.3. Study Variables

Variables analyzed included demographics (age, sex, and socioeconomic status), time to admission (from first visit at the unit to date of admission), overall comorbidity (assessed with the Charlson comorbidity index (1, 2, >3)) [31], performance status in patients with a new diagnosis of cancer according to the Eastern Cooperative Oncology Group Performance Status (ECOG-PS) scale (0–1 (absent/minor impairment), 2 (moderate impairment) and 3–4 (severe impairment)) [32], reasons for admission, and invasive diagnostic procedures performed.

### 2.4. Statistical Analysis

All data were entered prospectively in a database beginning in 2004. The χ^2^ test or the Fisher’s exact test, as appropriate, was used to compare categorical data, which are expressed as numbers and percentages. We used *t*-tests to compare continuous variables with a normal distribution, which are expressed as mean and standard deviation (SD), or the nonparametric Mann–Whitney *U* test to compare continuous variables with a skewed distribution, which are expressed as median and interquartile range (IQR).

Linear regression was used to test for time trends with yearly admissions as the dependent variable and the number of years as the independent variable. Trends were considered significant when the slope of the trend was not equal to zero and *p* < 0.05. The estimation of the linear trend slope provides a quantitative interpretation of the magnitude of the trend, that is, the mean yearly increase (positive slope) or decrease (negative slope) of the dependent or measured variable. All analyses were performed with GraphPad Prism v8 (GraphPad Software, San Diego, CA, USA).

## 3. Results

**Figure 1:**
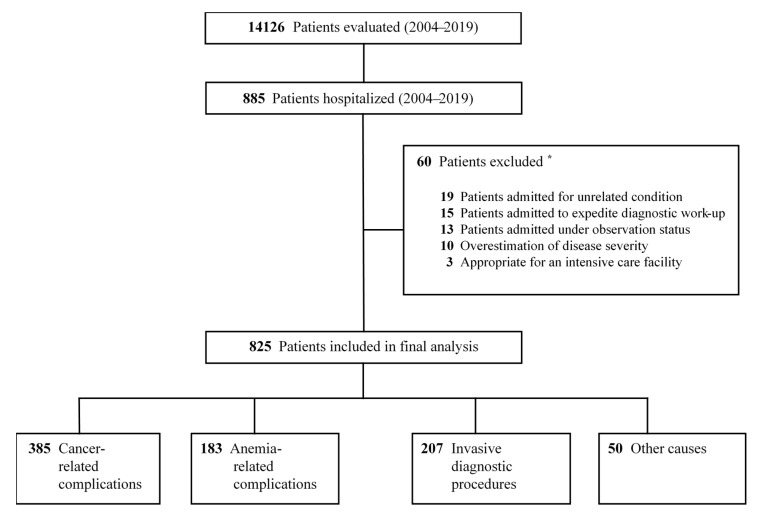
Flowsheet for the patient selection process. * According to independent assessment of appropriateness of admission decisions.

During 2004–2019 a total of 14,126 patients were evaluated at the quick diagnosis unit. Among these, we found 885 initially eligible patients who were hospitalized during their management at the unit. Based on experts’ review of appropriateness, 60 out of the 885 (6.8%) admissions were determined to be inappropriate and were excluded from the final study (Figure 1). The degree of overall agreement between the physician reviewers was 93.2%, with an agreement value of 94.3% for appropriate admissions and 91.2% for inappropriate admissions. The kappa coefficient for overall inter-rater agreement was 0.83 (95% confidence interval, 0.79–0.87; *p* < 0.05).

The characteristics of the 825 patients included in the study were compared each year and linear trends were analyzed. First, we examined the total number of patients evaluated at the unit for each-calendar year from 2004–2005 to 2018–2019 and analyzed the temporal trends in hospitalizations as a proportion of all patients managed at the unit. Next, we examined trends in patient demographics, Charlson comorbidity index, ECOG-PS score in cancer patients, time to admission, and major reasons for admission. We also calculated differences in the frequency of invasive procedures performed according to the specific type of procedure.

**Figure 2:**
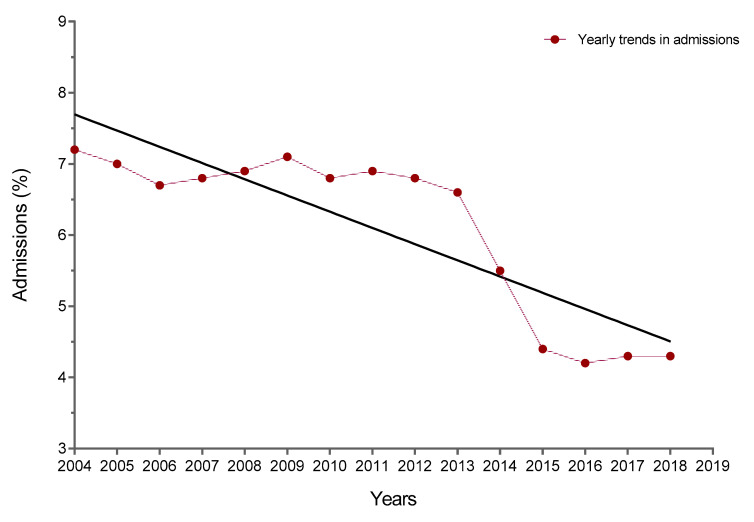
Temporal trends in admission rates for patients evaluated at the quick diagnosis unit from 2004–2005 to 2018–2019.

The number of hospitalizations decreased over time with a significant linear trend (Figure 2). The slope of annual change was significantly different from zero; it was −0.2278 per year (95% CI, −0.3094 to −0.1463; *p* < 0.0001). The admission rate was 7.2% in 2004–2005, 6.8% in 2012–2013, and 4.3% in 2018–2019 (Table 1). The median time to admission throughout the entire study period was 7.5 (6–9) days. For 2004–2005, the time to admission was 8 (6–9) days and it decreased to 6 (5–7) days in 2014–2015 and 5 (4–6) days in 2018–2019 (*p* < 0.0001).

Table 1 shows temporal trends in characteristics of patients hospitalized throughout 15 years. Overall, the mean age was 65.4 (16.1) years and 49.2% were women. Patients aged >65 years comprised 53.8% of the study population. Both age and gender distribution remained unchanged over time, as did the distribution of the income quartile. No significant yearly changes were observed in comorbidity according to the mean Charlson comorbidity index (1.81 (0.81) in 2004–2005 vs. 1.84 (0.83) in 2018–2019; *p* = 0.2867).

Based on a thorough assessment of the reasons for admission, four major categories were established: (1) cancer-related complications; (2) anemia-related complications; (3) invasive procedures; and (4) other causes (see Table 2 for details). Among 825 hospitalized patients, 433 (52.5%) were diagnosed with cancer and 385 (46.7%) were hospitalized for cancer-related complications. While the proportion of hospital admissions of patients with a diagnosis of cancer increased significantly over time, from 44.7% in 2004–2005 to 66.7% in 2018–2019 (*p* < 0.0001), a significant increasing trend was also observed in the rate of admissions due to cancer-related complications, from 39.5% in 2004–2005 to 53.5% in 2014–2015 to 61.7% in 2018–2019 (Table 1). We detected a significant increase in the number of cancer patients with a worse performance status (ECOG-PS scale, 2 to 4) over time. While the mean score in the ECOG-PS scale was 2.38 (1.36) in 2004–2005, it was 3.03 (1.65) in 2018–2019 (*p* < 0.0001). Indeed, a decline in performance status, frequently accompanied by other symptoms or added multimorbidity, was the main reason for admission in 338 of 385 (87.8%) patients with cancer-related complications during the time of the study.

**Figure 3:**
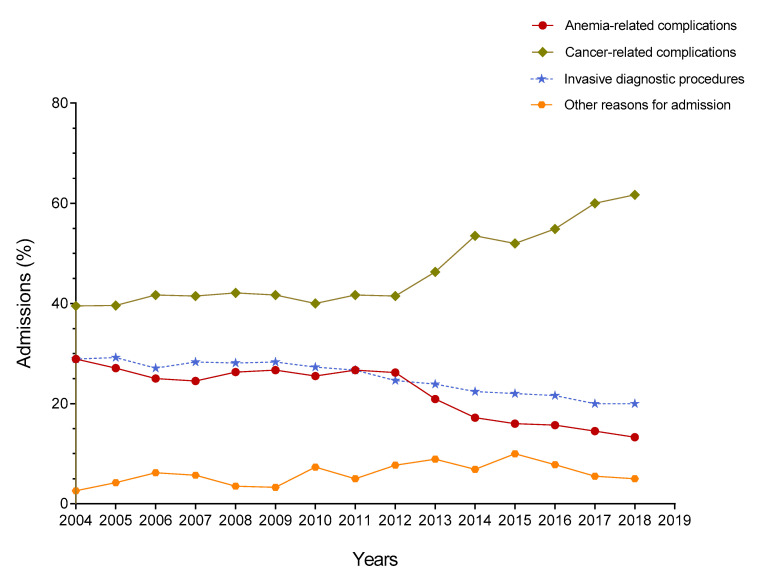
Trends in admissions according to the main categories of reasons for admission.

Anemia-related complications was the reason for admission in 183 of 825 (22.2%) patients. An analysis of yearly trends revealed a significant downward trend in the percent of these admissions, from 28.9% in 2004–2005 to 20.9% in 2013–2014 to 13.3% in 2018–2019 (*p* < 0.0001; Figure 3). The occurrence of heart failure and/or angina pectoris in older patients with severe anemia and comorbidities, mostly cardiovascular comorbidity, accounted for 167 of 183 (91.3%) admissions due to anemia-related complications.

Out of 825 patients, 207 (25.1%) were hospitalized for undergoing invasive diagnostic procedures and this rate decreased significantly from 28.9% during 2004–2005 to 22% during 2015–2016 to 20% in 2018–2019 (*p* < 0.0001; Figure 3). In all three major admission categories (cancer-related complications, anemia-related complications, and invasive procedures), the slopes of yearly change were significantly different from zero (*p* < 0.0001). A further 50 (6.1%) patients were hospitalized due to other causes.

To further analyze the trends in the frequency of invasive procedures, these were categorized into the following groups: computed tomography- or ultrasound-guided biopsy (liver, pancreas, lung/pleura, and bone), endoscopic retrograde cholangiopancreatography (ERCP), and other (arteriography and diagnostic/therapeutic surgery including laparoscopy and pleuroscopy). Most frequently performed procedures were liver biopsy and ERCP, while the joint proportion of pancreatic, lung/pleural, and bone biopsies was only 16.4%. As shown in Table 3, there was a significant increase in the frequency of liver biopsy (from 54.5% in 2004–2005 to 75% in 2018–2019; *p* = 0.0004) and ERCP (from 9.1% in 2004–2005 to 27.3% in 2017–2018; *p* = 0.0231). In contrast, the proportion of patients who were admitted for pancreatic, lung/pleural, and bone biopsies declined significantly over time.

## 4. Discussion

This investigation examined the trends in return hospitalizations of patients with serious illnesses managed at a hospital-based quick diagnosis unit over 15 years. Three key findings were reported in our study. Firstly, with a rate of admissions of 5.8% for the whole period, trends decreased significantly with an average yearly change of 2.3%; Secondly, the occurrence of cancer-related complications, mainly a decline in performance status, was the leading reason for hospitalizations for each-calendar year with a significant increasing trend from the first to the last period; Finally, these increasing trends were counterbalanced by significant decreases in the rates of hospitalizations for both anemia-related complications and invasive examinations.

As far as we can tell, there are no previous reports analyzing temporal trends including clinical characteristics and causes of hospitalizations for patients evaluated at quick diagnosis units. Evidence for the role of quick diagnosis units as an alternative ambulatory care model to inpatient admission comes mainly from level 2a and 2b studies, including two systematic reviews [11,33] and observational studies (prospective and retrospective) [16,17,18,19,20,24,25]. A systematic review reported in 2014 by independent US authors selected for final review four studies that focused on quick diagnosis units, of which three had been published in Spanish medical journals [33]. Another systematic review reported in 2016, also by independent US authors, reviewed all the systematic reviews about alternative strategies to inpatient admission for acute medical conditions including quick diagnosis units [11]. According to these studies, malignancy was the most common final diagnosis in patients managed at quick diagnosis units (18–30% of cases), time from initial contact to diagnosis ranged from 6 to 11 days, patient satisfaction with the model was high, and associated costs were significantly lower than hospitalization. Although the evidence was more limited, the associated mortality was low (0.3% in a large prospective trial of 4170 patients) [19] and two Spanish studies reported an admission rate of 7% and 10% [12,13]. A ‘poor general condition’ was mentioned in one report as the reason for admission without further details [13]. A model similar to quick diagnosis units, the Diagnostic Center, was implemented in Scandinavian countries in the early 2010s. Its aim is to provide an ‘urgent diagnostic pathway’ for patients referred from primary care with nonspecific symptoms and signs of serious disease, mostly cancer. Although studies of patients evaluated at these ambulatory care clinics have reported on different outcome indicators such as time intervals, impact on health-related quality of life, patient reported experience and survival rates, data of return hospitalizations or return visits are lacking [34,35,36,37,38].

A salient characteristic of our study was the inclusion of patients who required admission for invasive investigations, which represents an appropriate reason for admission according to the operational protocol of our hospital and the AEP [26,27,28,29]. Unlike the other admission categories, however, hospitalization in these cases is always planned. Had these admissions be excluded from the study protocol to focus only on unwanted ones, the average rate of admissions throughout 15 years would have been 4.4%.

Our results showed that trends in admission for anemia-related complications decreased sharply from 20.9% in 2013–2014 to 13.3% in 2018–2019. Furthermore, trends in admission for invasive procedures remained stable from 2006–2007 to 2010–2011 (27.1% to 27.3%, respectively) then decreased to 20% in 2018–2019. When looking at admission rates according to types of procedures, no admissions for bone, pancreatic, and lung/pleural biopsies were further registered after the time periods 2009–2010, 2012–2013, and 2013–2014, respectively. A likely explanation for these findings was the relocation of the quick diagnosis unit to the daycare center in 2013–2014. Unlike the original unit at the hospital outpatient department, this daycare center is fully staffed with physicians from different specialties and three full-time nurses. In addition to two recovery rooms for procedures requiring sedation or anesthesia and a post-procedure observation time, six armchairs are used for blood transfusions and intravenous treatments such as intravenous iron. This daycare center hence offered the chance to manage more efficiently ambulatory patients with severe anemia, mainly iron-deficiency anemia, who otherwise would have been admitted due to complications such as acute heart failure [39]. Similarly, the availability of recovery rooms meant a shift in the need for inpatient beds for invasive examinations including pancreas, lung/pleural, and bone biopsies. However, due to a higher risk of procedure-related complications, liver biopsies and ERCP are still procedures requiring admission to our hospital for the day of the procedure.

As our results provide evidence that inpatient admissions represent an underreported unfavorable outcome in patients managed at quick diagnosis units, we can envisage some potential implications for clinical practice and hospital management. In our experience, cancer-related complications, mostly an impaired performance status, was the foremost cause of unplanned admissions for the whole duration of the study. The performance status of patients admitted with a new diagnosis of cancer worsened progressively over time, with 44.1% scoring 3 or 4 points in 2004–2005 compared to 55% in 2018–2019 (*p* < 0.0001). Understanding and anticipating this risk in patients in whom cancer is suspected and eventually diagnosed may assist in a quick delivery of optimal care. Furthermore, the potential impact of the daycare center on the declining trends in admissions for anemia-related complications suggests that patients referred to quick diagnosis units for investigation of severe anemia can be best screened and managed in this type of ambulatory care center. Previous reports have shown that ultrasound and computed tomography-guided biopsies, such as those described here, can be safely performed on an outpatient basis and that outpatient observation without hospitalization is also safe for liver biopsies [40,41,42,43]. The decreasing trends in admissions for pancreatic, lung/pleural, and bone biopsies around and after the relocation of the quick diagnosis unit to the daycare center, with availability of recovery rooms for post-procedure observation also support this care delivery resource for these patients.

### Strengths and Limitations of This Study

The 15 year duration of the study with an analysis of the changing admission trends for the whole population of patients managed at the quick diagnosis unit since its creation in 2004 is one of its strengths. In addition, although admission trends were analyzed collectively, the separate categorization of reasons for admissions according to disease-related complications and planned admissions for invasive procedures allowed a clearer interpretation of the main findings of the study. Nonetheless, it should be interpreted in the context of its limitations. First, data were collected from a single, academic medical center and might not be generalizable to all other quick diagnosis units; Second, although detailed clinical information of all patients was carefully reviewed, some relevant facts might not have been completely captured and errors could exist in patient recall and documentation—a limitation related to the retrospective design; Finally, there may be other unidentified factors that could have influenced return hospitalizations during the 15 year study period. However, aside from the daycare center, there were no other changes in facilities or staffing. Likewise, it may be argued that the lack of a non-intervention group to which hospitalization trends could be compared does not fully answer the question of whether the observed trends were attributable to the quick diagnosis unit only or to other external factors.

## 5. Conclusions

Our research found that overall trends in return hospitalizations of patients with serious conditions evaluated at a quick diagnostic clinic decreased significantly over 15 years at an average annual rate of 2.3%. An analysis of different variables showed that age, gender, socioeconomic status, and comorbidities did not have a relevant impact on these trends. While yearly admission trends increased significantly for complications associated with a new diagnosis of cancer, most notably an impaired performance status, trends declined in a significant manner during the second half of the study for hospitalizations for anemia-related complications and invasive diagnostic investigations. The availability of a new daycare center with armchairs for intravenous treatments and recovery rooms for post-procedure observation around the middle of the period of the study was a relevant factor contributing to these downward trends. Our characterization of trends in return hospitalizations could help clinicians identify when patients with serious diseases are at higher risk of clinical complications and provide anticipatory guidance for their best management. In our view, despite the limitations outlined, the facts of this investigation might also encourage the development of policies aimed at promoting daycare centers for the management of patients at quick diagnosis units.

## Figures and Tables

**Table 1:** Trends in characteristics for patients hospitalized from 2004 to 2019.

Characteristics	Overall	2004–2005	2005–2006	2006–2007	2007–2008	2008–2009	2009–2010	2010–2011	2011–2012	2012–2013	2013–2014	2014–2015	2015–2016	2016–2017	2017–2018	2018–2019	*p*-Value (Trend)
Total population, *N*	14,126	523	689	720	775	832	849	812	872	949	1021	1055	1140	1224	1281	1384	
Admissions, *n* (%)	825 (5.8)	38 (7.2)	48 (7.0)	48 (6.7)	53 (6.8)	57 (6.9)	60 (7.1)	55 (6.8)	60 (6.9)	65 (6.8)	67 (6.6)	58 (5.5)	50 (4.4)	51 (4.2)	55 (4.3)	60 (4.3)	<0.0001
Age, years, mean (SD)	65.4 (16.1)	64.6 (12.5)	65.7 (14.1)	64.8 (12.9)	66.0 (14.0)	62.7 (13.6)	67.3 (14.9)	62.9 (15.1)	68.4 (15.0)	65.9 (14.4)	63.6 (15.3)	67.5 (13.9)	66.8 (14.2)	68.1 (13.7)	64.2 (15.5)	63.3 (16.0)	0.1165
Age ≥ 65 years, %	53.8	53.2	54.1	53.3	54.3	51.6	55.4	51.8	56.3	54.2	52.3	55.6	55.0	56.0	52.8	52.1	0.1033
Female sex, *n* (%)	406 (49.2)	17 (44.7)	23 (47.9)	25 (52.1)	26 (49.1)	30 (52.6)	27 (45)	27 (49.1)	29 (48.3)	33 (50.8)	32 (47.8)	29 (50.0)	26 (52.0)	25 (49.1)	27 (49.1)	30 (50.0)	0.0755
Socioeconomic status quartile, *n* (%) *																	0.1445
Quartile 1	116 (14.1)	5 (13.2)	7 (14.6)	7 (14.6)	8 (15.1)	9 (15.8)	9 (15.0)	7 (12.7)	8 (13.3)	9 (13.8)	9 (13.4)	7 (12.1)	6 (12.0)	6 (11.8)	9 (16.4)	10 (16.7)	
Quartile 2	212 (25.7)	10 (26.3)	13 (27.1)	14 (29.2)	14 (26.4)	15 (26.3)	16 (26.7)	14 (25.4)	15 (25.0)	16 (24.6)	15 (22.4)	14 (24.1)	13 (26.0)	13 (25.5)	15 (27.3)	15 (25.0)	
Quartile 3	274 (33.2)	12 (31.6)	15 (31.3)	14 (29.2)	16 (30.2)	18 (31.6)	19 (31.7)	19 (34.5)	21 (35.0)	23 (35.4)	26 (38.8)	20 (34.5)	18 (36.0)	18 (35.3)	17 (30.9)	18 (30.0)	
Quartile 4	223 (27.0)	11 (28.9)	13 (27.1)	13 (27.1)	15 (28.3)	15 (26.3)	16 (26.7)	15 (27.3)	16 (26.7)	17 (26.2)	17 (25.4)	17 (29.3)	13 (26.0)	14 (27.4)	14 (25.4)	17 (28.3)	
Charlson index, mean (SD)	1.88 (1.12)	1.81 (0.81)	1.84 (0.92)	1.90 (1.01)	1.94 (1.12)	1.76 (0.87)	1.98 (1.23)	1.70 (0.88)	2.04 (1.19)	2.20 (1.20)	1.78 (1.07)	1.92 (0.95)	1.88 (1.00)	1.96 (1.14)	1.73 (0.99)	1.84 (0.83)	0.2867
0–1, *n* (%)	275 (33.3)	14 (36.8)	17 (35.4)	16 (33.3)	17 (32.1)	21 (36.8)	19 (31.7)	20 (36.4)	18 (30.0)	19 (29.2)	24 (35.8)	19 (32.8)	16 (32.0)	15 (29.4)	19 (34.5)	21 (35.0)	
2, *n* (%)	365 (44.2)	15 (39.5)	19 (39.6)	20 (41.7)	22 (41.5)	25 (43.9)	27 (45.0)	25 (45.5)	27 (45.0)	29 (44.6)	29 (43.3)	26 (44.8)	24 (48.0)	25 (49.0)	26 (47.3)	26 (43.3)	
>3, *n* (%)	185 (22.4)	9 (23.7)	12 (25.0)	12 (25.0)	14 (26.4)	11 (19.3)	14 (23.3)	10 (18.2)	15 (25.0)	17 (26.2)	14 (20.9)	13 (22.4)	10 (20.0)	11 (21.6)	10 (18.2)	13 (21.7)	
Cancer patients, *n* (%)	433 (52.5)	17 (44.7)	22 (45.8)	23 (47.9)	26 (49.1)	27 (47.4)	29 (48.3)	25 (45.5)	29 (48.3)	30 (46.2)	35 (52.2)	34 (58.6)	28 (56.0)	31 (60.8)	37 (67.3)	40 (66.7)	<0.0001
ECOG-PS score mean (SD)	2.78 (1.84)	2.38 (1.36)	2.60 (1.45)	2.49 (1.51)	2.59 (1.52)	2.49 (1.60)	2.67 (1.55)	2.70 (1.49)	2.79 (1.72)	2.81 (1.40)	2.79 (1.67)	2.87 (1.81)	2.89 (1.73)	2.94 (1.86)	3.01 (1.58)	3.03 (1.65)	<0.0001
0–1, *n* (%)	77 (17.8)	5 (29.4)	5 (22.7)	6 (26.1)	6 (23.1)	7 (25.9)	6 (20.7)	5 (20.0)	5 (17.2)	5 (16.7)	6 (17.1)	5 (14.7)	4 (14.2)	4 (12.9)	4 (10.8)	4 (10.0)	
2, *n* (%)	165 (38.1)	6 (35.3)	9 (40.9)	9 (39.1)	11 (42.3)	10 (37.0)	12 (41.4)	10 (40.0)	12 (41.4)	12 (40.0)	13 (37.1)	13 (38.2)	10 (35.7)	11 (35.5)	13 (35.1)	14 (35.0)	
3–4, *n* (%)	191 (44.1)	6 (35.3)	8 (36.3)	8 (34.8)	9 (34.6)	10 (37.0)	11 (37.9)	10 (40.0)	12 (41.4)	13 (43.3)	16 (45.7)	16 (47.1)	14 (50.0)	16 (51.6)	20 (54.1)	22 (55.0)	
Time-to-admission, days, median (IQR)	7.5 (6–9)	8 (6–9)	9 (7–10)	8 (7–10)	10 (8–11)	8 (7–9)	10 (8–12)	9 (8–10)	8 (6–9)	7 (5–8)	7 (6–8)	6 (5–7)	7 (6–8.5)	5 (4–7)	6 (5–7)	5 (4–6)	<0.0001

* Quartile of the mean household income: lowest = quartile 1, low–middle = quartile 2, high–middle = quartile 3, highest = quartile 4. Trends for lowest income quartile vs. others. SD, standard deviation; ECOG-PS, Eastern Cooperative Oncology Group Performance Status; IQR, interquartile range.

**Table 2:** Categories of reasons for admissions.

**Cancer-Related Complications**
Decline in performance status
Unmanageable severe pain
Other
Thromboembolic disease
Hypercalcemia
Superior vena cava syndrome
Brain metastases
Spinal cord compression
**Anemia-Related Complications**
Cardiovascular complications
Unresponsive severe anemia/worsening anemia
Recurrent severe anemia
Other
Invasive Procedures *
Computed tomography- or ultrasound-guided biopsy
Liver
Pancreas
Lung/pleura
Bone
Endoscopic retrograde cholangiopancreatography
Other
Diagnostic and/or therapeutic surgical procedures including laparoscopy and pleuroscopy
Arteriography
**Other Causes**
Biliary obstruction
Active bleeding
Malnutrition/starvation
Severe electrolyte imbalance
Respiratory complications
Need for fluid replacement or intravenous medications
Infections
Infective endocarditis
Tuberculosis
Spondylodiscitis
Visceral leishmaniasis
Other

* Invasive studies require admission to the hospital for the day of the procedure.

**Table 3:** Trends in invasive procedures for patients hospitalized from 2004 to 2019.

Type of Procedure	Overall	2004–2005	2005–2006	2006–2007	2007–2008	2008–2009	2009–2010	2010–2011	2011–2012	2012–2013	2013–2014	2014–2015	2015–2016	2016–2017	2017–2018	2018–2019	*p*-Value (Trend)
Admissions, *n*	825	38	48	48	53	57	60	55	60	65	67	58	50	51	55	60	
Procedure, *n* (%)	207 (25.1)	11 (28.9)	14 (29.2)	13 (27.1)	15 (28.3)	16 (28.1)	17 (28.3)	15 (27.3)	16 (26.7)	16 (24.6)	16 (23.9)	13 (22.4)	11 (22)	11 (21.6)	11 (20)	12 (20)	<0.0001
Liver biopsy	132 (63.8)	6 (54.5)	6 (42.9)	8 (61.5)	8 (53.3)	9 (56.3)	9 (52.9)	10 (66.7)	11 (68.8)	12 (75.0)	11 (68.8)	9 (69.2)	9 (81.8)	7 (63.6)	8 (72.7)	9 (75.0)	0.0004
Pancreatic biopsy	17 (8.2)	2 (18.2)	2 (14.3)	2 (15.4)	3 (20)	3 (18.8)	2 (11.8)	1 (6.7)	1 (6.3)	1 (6.3)	0 (0)	0 (0)	0 (0)	0 (0)	0 (0)	0 (0)	<0.0001
Lung/pleural biopsy	12 (5.8)	1 (9.1)	2 (14.3)	1 (7.7)	1 (6.7)	2 (12.5)	2 (11.8)	1 (6.7)	1 (6.3)	0 (0)	1 (6.3)	0 (0)	0 (0)	0 (0)	0 (0)	0 (0)	<0.0001
Bone biopsy	5 (2.4)	1 (9.1)	1 (7.1)	0 (0)	1 (6.7)	1 (6.3)	1 (5.9)	0 (0)	0 (0)	0 (0)	0 (0)	0 (0)	0 (0)	0 (0)	0 (0)	0 (0)	0.0013
ERCP	33 (15.9)	1 (9.1)	2 (14.3)	1 (7.7)	2 (13.3)	1 (6.3)	2 (11.8)	2 (13.3)	3 (18.8)	2 (12.5)	4 (25.0)	3 (23.1)	2 (18.2)	3 (27.3)	3 (27.3)	2 (16.7)	0.0231
Other *	8 (3.9)	0 (0)	1 (7.1)	1 (7.7)	0 (0)	0 (0)	1 (5.9)	1 (6.7)	0 (0)	1 (6.3)	0 (0)	1 (7.7)	0 (0)	1 (9.1)	0 (0)	1 (8.3)	0.1213

* Arteriography and diagnostic and/or therapeutic surgical procedures including laparoscopy and pleuroscopy. ERCP, endoscopic retrograde cholangiopancreatography.

## Supplementary Materials

The following are available online at https://www.mdpi.com/2075-4418/10/8/585/s1, Figure S1: Appropriateness Evaluation Protocol-based chart form used to determine the appropriateness of admissions including justification of appropriateness and reason for inappropriateness.
