# Supplementary material for: Trends in Hospitalization of Patients with Potentially Serious Diseases Evaluated at a Quick Diagnosis Clinic
Source: Diagnostics (Basel). 2020 Aug 13;10(8):585. doi: 10.3390/diagnostics10080585 (PMC7460236; doi:10.3390/diagnostics10080585)
Supplement: Supplementary file 1 [file diagnostics-10-00585-s001.pdf]

APPROPRIATENESS OF ADMISSION CHART FORM

Reviewer's name (full initials):

Hospital record #

Date of review:

SOCIODEMOGRAPHIC DATA

Age: years **Gender**: 1. Male 2. female

Location at time of admission:

Residential location: 1. Urban 2. Rural 3. Not specified

Location name:

Living alone: 1. Yes 2. No 3. Not specified

GENERAL INFORMATION

Referred from: 1. Emergency 2. Primary Care 3. Outpatients 4. Other

Date of first appointment at quick diagnosis unit: **Date of hospital admission**:

Type of admission: 1. Unplanned (urgent) 2. Unplanned (prescheduled) 3. Planned

In case of *planned admission*, was it arranged to perform an invasive procedure?

1. Yes 2. No

Please specify *type of procedure*:

Department where patient was admitted:

Specialty (specify):

CLINICAL DATA

Patient general status at time of admission (select a choice):

1. Asymptomatic
2. Symptomatic but completely ambulatory
3. Symptomatic, <50% in bed during the day
4. Symptomatic, >50% in bed
5. Bedbound (completely disabled)

Diagnosis (presumptive or definitive) at time of admission:

Primary:

Secondary:

Reason for admission (multiple choices allowed):

1. Cancer-related complications
2. Anemia-related complications
3. Invasive procedure
4. Other (specify)*

**Was comorbidity a contributing factor to admission decision?* 1. Yes 2. No*

Diagnosis at time of hospital discharge:

Primary:

Secondary:

APPROPRIATENSS OF ADMISSION

Admission decision according to appropriateness of admission criteria:

1. Appropriate
2. Inappropriate
3. Unclear/difficult to determine (specify)

Justification for appropriateness of admission (multiple choices allowed):

1. Severity of illness
2. Intensity of service
3. Other (specify)

Reason for inappropriate admission (multiple choices allowed):

1. Admission to avoid the waiting time for an outpatient investigation
2. Overestimation of illness severity
3. Inappropriate level of care
4. Admission claimed by patient or family to avoid paying for certain costs
5. Social problems
6. Previous consultation would be required to make a decision on admission
